# Guarana in Headache

**Published:** 1874-11

**Authors:** 


Guarana in Headache.—The following case is reported in the Canada *Medical Record*, by Dr. James Perrigo, Montreal: H. R., a wealthy merchant of this city, suffered fearfully from severe headaches. He was liable to be attacked at any time of the day, and most particularly on those days when business cares pressed more heavily upon him. His digestive organs were in good condition, and he never had suffered from constipation or any of the many different forms of dyspepsia. Among articles of diet, stimulants alone would bring on the headache. He could not read an article, however light and amusing, without immediately suffering. The pain extended over the temporal and occipital regions, and down the neck, not following the course of any particular nerve. Never felt any nausea during an attack. Artificial light of any kind, either in his study or store, caused the pain to be intense, and then he only felt it on the crown of his head. If he bowed his body to pick up anything from the floor, it was as much as he could do to regain the erect posture. Sometimes the pain was so agonizing that he was obliged to lie down from sheer inability to hold up his head.

Previous to his coming under my care, he had been leeched, blistered repeatedly behind the ears; had been ordered bromide potassium, valerianate of ammonia, iodide of potassium, quinine, without deriving even temporary benefit; and he had also given homœopathy a fair trial. At last he was obliged to absent himself from business, when he went into the country for a couple of months, and returned much better. A month after reapplication to business, the headaches returned, but not so severe as formerly. This was six months ago. Lately, however, their severity has been increasing, and he says they are nearly as bad as ever. A mutual friend advised him to come to me for electrical treatment, and this is the history I elicited from him. Hearing so much about the wonderful effects of guarana in kindred cases, I expressed my wish to give it a trial. He consented, and I prescribed thirty grains of the powder in water, to be taken when the pain was severe. It acted exceedingly well, completely relieving him of all his headache. At present he can invariably prevent an attack by taking the above dose when he feels the premonitory symptoms coming on.

